# Prevalence of Diabetes and Cardiovascular Comorbidity in the Canadian Community Health Survey 2002–2003

**DOI:** 10.1100/tsw.2006.13

**Published:** 2006-01-24

**Authors:** Frank Mo, Lisa M. Pogany, Felix C.K. Li, Howard Morrison

**Affiliations:** Centre for Chronic Disease Prevention and Control, Public Health Agency of Canada, Government of Canada, 120 Colonnade Road, Ottawa, Ontario, K1A 0K9, Canada

**Keywords:** diabetes, comorbidity, hypertension, stroke, heart disease, risk factors, lifestyle, social economics, health survey, epidemiology, public health, human development, Canada

## Abstract

Diabetes mellitus is a major risk factor for heart disease (heart attack, angina, and heart failure), stroke, and hypertension, which shorten the average life expectancy. The main objective of this study was to describe the prevalence of heart disease, hypertension, and stroke among Canadians with diabetes compared to those without diabetes in the Canadian general population aged 12 years and over. It also estimated the strength of association between diabetes, heart disease, hypertension, and other factors such as age, gender, cigarette smoking, alcohol drinking, education status, body mass index (BMI), and other socioeconomic factors. Descriptive statistics were used initially to estimate the prevalence of related comorbidities by age and gender. Logistic regression was then employed to determine the potential strength of association between various effects. Data included 127,610 individuals who participated in the 2.1 cycles of the Canadian Community Health Survey (CCHS) in 2002–2003. The prevalence of self-reported hypertension, heart disease, and stroke among individuals with diabetes were 51.9, 21.7, and 4.8%, respectively. By comparison, prevalence among those without diabetes was 12.7, 4.2, and 0.9%. Adjusted Odds Ratios (OR) were 4.15, 5.04, and 6.75 for males, and 4.10, 5.29, and 4.56 for females hypertension, heart disease, and stroke, respectively. Lower income (OR from 1.27—1.94) and lower education (OR from 1.23—1.86) were independently associated with a high prevalence of hypertension, heart disease, and stroke among diabetics. Alcohol consumption (OR from 1.06—1.38), high BMI (OR from 1.17—1.40), physical inactivity (OR from 1.21—2.45), ethnicity, and immigration status were also strongly associated with hypertension, heart disease, and stroke. The adjusted prevalence of hypertension, heart disease, and stroke in the CCHS-2003 health survey in Canada was significantly higher among those with diabetes compared to those without. Other factors such as age, gender, BMI, lifestyle, family incomes, physical activity levels, and socioeconomic status also affected the strength of association between diabetes and resulting comorbidities.

### INTRODUCTION

Diabetes mellitus is the seventh leading cause of death in Canada, affects more than 2 million Canadians, and may shorten life expectancy by up to 15 years[1,2]. The risk for heart disease and stroke among individuals with diabetes, compared to those without, is about four- to sixfold higher and approximately 50% of adults with diabetes have high blood pressure[3]. In a recent study from Germany, severe complications were diagnosed in 50% of diabetes patients including myocardial infarction, stroke, foot ulcer, amputations, and blindness[4].

The lifetime costs associated with comorbidities of diabetes mellitus are substantial in Canada; the cost of diabetes was estimated to be $1.6 billion in 1998, an increase of 45% from the estimate of $1.1 billion in 1993[5] and is likely to be $19.2 billion in 2020[6]. Direct medical costs of managing complications from diabetes average $47,240 per patient over 30 years (in 2000 U.S. dollars)[7]. More severe or debilitating events generate a greater financial burden than do early-stage complications and complications that are initially relatively low in cost can progress to more costly advanced stages[8]. This significant reduction of life span and quality of life, along with the financial costs of diabetes, suggest that action is required for prevention and control of complications of diabetes[1].

The overall picture of cardiovascular comorbidities of diabetes in the general Canadian population is likely to be similar to other developed countries, however, there have not been specific Canadian population estimates among age and sex subpopulations. Further, the role of health behaviors and socioeconomic status on cardiovascular comorbidities is unknown. Evidence-based guidance for control and management in the context of multiple comorbid conditions and complications in hospital settings is required.

The objectives of this study were to describe self-reported comorbidities among diabetics compared to nondiabetics using the Canadian Community Health Survey (CCHS) of 2002–2003, and to assess the impact of health behaviors and socioeconomic status on cardiovascular comorbidities.

### METHODS

#### Canadian Community Health Survey

The Canadian Community Health Survey (CCHS) of 2002–2003[9] is a collaborative effort between Statistics Canada, Health Canada, and the Canadian Institute for Health Information. This survey was created to gather health-related data at subprovincial levels in order to provide timely cross-sectional estimates of health determinants, health status, and health system utilization. The CCHS began collecting data in September of 2000 and the data collection was based on a 2-year collection cycle.

#### Sampling

Each of the collection cycles is comprised of two distinct surveys: a health region–level survey in the first year with a total sample of 130,000 and a provincial-level survey in the second year with a total sample of 30,000. The target population for the CCHS 2003 was household residents in all Canadian provinces and territories; with the exclusion of Canadian Forces Bases and some remote areas. One member of each household sample was selected with a second member of certain households being sampled in order to oversample for youths.

#### Measures of Diabetes and Comorbidity

Diabetes was identified by self-report based on the question “Has your doctor ever said that you had diabetes?” The comorbidities, also diagnosed by self-report, were in response to the question “Has

(comorbidity) lasted or expected to last 6 months or more and was it diagnosed by a health professional?" The comorbidities inquired about by the CCHS-2003 questionnaire included allergy, asthma, arthritis or rheumatism, back problem, high blood pressure, migraine headaches, chronic bronchitis or emphysema, sinusitis, epilepsy, heart disease, cancer, stomach or intestinal ulcers, effects of a stroke, urinary incontinence, bowel disorder, dementia, cataracts, glaucoma, and thyroid condition.

### Measures of Diabetes Covariate Variables

The self-reported covariates included in this analysis were age, gender, area of residence, body mass index (BMI), physical activity, socioeconomic status (income, ethnicity, immigration, education), smoking status (former smokers, current smokers, and never smokers), and heavy alcohol intake. BMI was calculated from the equation BMI = weight (kg)/height (m)$^2$. Physical activity was grouped as active, moderate, and inactive based on leisure time energy expenditure[5]. Education was grouped into three levels based on the years of education individual gains: less than secondary school education, which indicated not finishing 12 years of basic education; secondary school education completed; and postsecondary school education. Physically active was defined as an energy expenditure of at least 3 KKD (kilocalories per kilogram of body weight per day), moderately active corresponds to an energy expenditure between 1.5 and 3 KKD, while physically inactive is defined as less than 1.5 KKD. Income adequacy was classified into low, low middle, upper middle, and high-income groups on the basis of total household income and the number of household members[10,11]. Ethnicity included Canadian and other minority origins. Immigration status includes Canadians that were born in Canada or immigrated from overseas. These definitions have been used in previous studies of similar Canadian data[12,13,14,15,16,17].

### Data Analysis

Descriptive statistics were used initially to estimate the prevalence of diabetes and its related comorbidities by age and gender. Logistic regression was then employed to determine the potential strength of association between various effects. Data included 127,610 individuals who participated in the 2.1 cycle of the Canadian Community Health Survey (CCHS) in 2002–2003. The logistic regression models were adjusted in order to take the design effects of the CCHS-2003 into account. These techniques have been used by in previous analyses of the national hospitalization data[18] and the NPHS data[12,13,14,15,16,17].

## RESULTS

The overall prevalence of self-reported diabetes mellitus in the CCHS-2003 cohort was 4.9% for males and 4.3% for females (Table 1), and the risk of diabetes was higher among males than females (Table 2). The prevalence of diabetes increased with increasing age and was most prevalent in those individuals over 65 years of age (13.5% for both sexes combined [Table 1]).

The cardiovascular comorbidities of hypertension, heart disease, and stroke were more prevalent among diabetics compared to nondiabetics (51.9 vs. 12.7% [OR = 4.12; 95% CI 4.05–4.19], 21.7 vs. 4.2% [OR = 5.16; 95% CI 5.12–5.23], and 4.8 vs. 0.9% [OR = 5.99; 95% CI 5.91–6.13], respectively [Table 2]). (OR, Odds Ratio; CI, Confidential Interval)

**TABLE 1**
**Distribution of Personal, Risk Factors, and Socioeconomic Characteristics by Diabetic Status Among Canadians 12 Years of Age and Over (CCHS, Canada, 2003)***

| | Diabetic Sample (n = 7,353) | | Nondiabetic Sample (n = 120,149) | |
|---|---|---|---|---|
| | n | % | n | % |
| **Sex** | | | | |
| Males | 3,521 | 4.9 | 54,892 | 95.1 |
| Females | 3,832 | 4.3 | 65,257 | 95.7 |
| **Age group (years)** | | | | |
| 12–34 | 338 | 0.7 | 42,261 | 99.3 |
| 35–64 | 3,335 | 4.9 | 54,984 | 95.1 |
| 65 and over | 3,680 | 13.5 | 22,904 | 86.5 |
| **Overweight or obese (BMI)** | | | | |
| =<25 | 2,023 | 28.0 | 67,929 | 64.8 |
| 26–29 | 1,789 | 32.2 | 23,321 | 21.4 |
| =>30 | 2,652 | 39.8 | 16,672 | 13.8 |
| **Family Income** | | | | |
| High income quartile | 1,272 | 22.3 | 12,416 | 37.6 |
| Upper middle income quartile | 2,017 | 33.0 | 22,255 | 34.5 |
| Low middle income quartile | 1,974 | 30.4 | 36,067 | 19.2 |
| Low income quartile | 1,053 | 14.2 | 31,614 | 8.8 |
| **Immigrant Status** | | | | |
| Immigrants | 1,102 | 26.2 | 15,101 | 20.2 |
| Non mmigrants | 6,119 | 73.8 | 103,075 | 79.8 |
| **Physical activity** | | | | |
| Active | 1,158 | 17.5 | 32,573 | 27.5 |
| Moderate | 1,495 | 22.1 | 29,285 | 25.2 |
| Inactive | 4,392 | 60.4 | 55,798 | 47.3 |
| **Education Level** | | | | |
| Postsecondary | 3,713 | 62.3 | 82,923 | 78.7 |
| Secondary | 974 | 13.4 | 16,827 | 12.4 |
| Less than secondary | 2,361 | 24.3 | 19,254 | 8.9 |
| **Heavy alcohol drinking** (once per week or everyday) | | | | |
| Yes | 1,713 | 57.2 | 44,603 | 48.0 |
| No | 2,667 | 42.8 | 45,897 | 52.0 |
| **Smoker Status** | | | | |
| Never | 2,032 | 16.1 | 42,664 | 37.5 |
| Former | 4,035 | 52.7 | 48,146 | 39.0 |
| Current | 1,265 | 31.2 | 28,991 | 23.5 |
| **Ethnicity** | | | | |
| First Nations | 1,550 | 19.3 | 25,514 | 20.1 |
| English origin | 1,518 | 17.5 | 22,618 | 16.3 |
| French origin | 1,201 | 13.8 | 19,821 | 15.1 |
| Other origin | 3,084 | 49.4 | 52,196 | 48.5 |

* Counts unweighted; percent weighted.

**TABLE 2**
**Prevalence (%) of Self-Reported Cardiac Comorbidities Among Diabetes Compared to Nondiabetics by Sex Among Canadians 12 Years of Age and Over (CCHS, Canada, 2003)**

| Diabetes Status | With Diabetes | | Without Diabetes | | Diabetes Status Difference | |
|---|---|---|---|---|---|---|
| | n | %* | n | %* | OR** | 95% CI |
| **Hypertension (Yes)** | | | | | | |
| Overall | 4,044 | 52.1 | 18,618 | 12.7 | 4.12 | 4.05–4.19 |
| Males | 1,749 | 48.5 | 7,337 | 11.7 | 4.15 | 4.10–4.21 |
| Females | 2,295 | 55.7 | 11,281 | 13.6 | 4.10 | 4.04–4.16 |
| **Heart disease (Yes)** | | | | | | |
| Overall | 1,719 | 21.7 | 6,802 | 4.2 | 5.16 | 5.12–5.23 |
| Males | 842 | 23.2 | 3,307 | 4.6 | 5.04 | 4.91–5.14 |
| Females | 877 | 20.1 | 3,495 | 3.8 | 5.29 | 5.20–5.38 |
| **Stroke (Yes)** | | | | | | |
| Overall | 399 | 4.8 | 1,340 | 0.8 | 5.99 | 5.91–6.13 |
| Males | 200 | 5.4 | 611 | 0.8 | 6.75 | 6.67–6.83 |
| Females | 199 | 4.1 | 729 | 0.9 | 4.56 | 4.47–4.68 |

* Percent weighted. ** Controlled for age.

### Relationship Between Gender on Cardiovascular Comorbidities

Female diabetics were more likely than male diabetics to report hypertension, but male diabetics were more likely than females to report heart disease or stroke (Table 2). Among males and females, respectively, hypertension was reported by half of diabetics (48.5 vs. 55.7%, respectively) compared to 11.7 and 13.6% of nondiabetics, respectively (OR = 4.15; 95% CI 4.13–4.17 and OR = 4.10; 95% CI 4.08–4.12 for males and females, respectively). Overall, female diabetics were more likely to report hypertension than male diabetics (OR = 1.14; 95% CI 1.12–1.15).

Heart disease was reported less frequently than hypertension at under one-quarter of male and female diabetics compared to less than 5% of their peers (OR = 5.04; 95% CI 5.01–5.08 and OR = 5.29; 95% CI 5.26–5.31 for males and females, respectively). However, once adjusted for age, females were more likely to report awareness of heart disease (OR = 1.15; 95% CI 1.13–1.16) (Table 2).

Lastly, stroke was more common among male and female diabetics compared to their peers (5.4 vs. 0.8% [OR = 6.75; 95% CI 6.72–6.79] and 4.1 vs. 0.9% [OR = 4.56; 95% CI 4.53–4.59], respectively). Overall, male diabetics were more likely to report a history of stroke compared to female diabetics (OR = 1.32; 95% CI 1.30–1.35) (Table 2).

### Relationship Between Health Behaviors on Cardiovascular Comorbidities

Among diabetics, the odds of reporting a cardiovascular comorbidity varied by a number of known risk factors. Compared to diabetics reporting a healthy BMI (≤25), those reporting increased BMI (≥30) also reported hypertension (42.4 vs. 59.5% [OR = 1.40; 95% CI 1.38–1.43]), heart disease (29.3 vs. 40.9% [OR = 1.17; 95% CI 1.15–1.19]), and stroke (4.1 vs. 5.1% [OR = 1.24; 95% CI 1.22–1.26]) more frequently (Table 3). Similarly, all cardiovascular comorbidities were reported more frequently among diabetics who reported alcohol consumption (OR from 1.06–1.38), current and former smokers (OR from 1.12–1.38), and those who were physically inactive (OR from 1.21–2.45) (Table 3).

**TABLE 3**
**OR and 95% CI of Awareness of Cardiac Comorbidities Among Diabetics by Risk Factors Among Canadians 12 Years of Age and Over (CCHS, Canada, 2003)***

| | Hypertension | | | | Heart Disease | | | | Stroke | | | |
|---|---|---|---|---|---|---|---|---|---|---|---|---|
| | n | % | OR | 95% CI | n | % | OR | 95% CI | n | % | OR | 95% CI |
| **BMI** | | | | | | | | | | | | |
| =<25 | 935 | 42.4 | ***1.00*** | — | 451 | 29.3 | ***1.00*** | — | 111 | 4.1 | ***1.00*** | — |
| 26-29 | 978 | 52.2 | 1.23 | 1.21–1.25 | 452 | 34.8 | 1.13 | 1.12–1.14 | 97 | 4.2 | 1.02 | 1.01–1.03 |
| =>30 | 1,650 | 59.5 | 1.40 | 1.38–1.43 | 592 | 40.9 | 1.17 | 1.15–1.19 | 136 | 5.1 | 1.24 | 1.22–1.26 |
| **Heavy alcohol drinking** | | | | | | | | | | | | |
| No | 887 | 48.7 | ***1.00*** | — | 324 | 16.2 | ***1.00*** | — | 71 | 3.8 | ***1.00*** | — |
| Yes | 1,440 | 50.6 | 1.04 | 1.03–1.06 | 565 | 19.2 | 1.19 | 1.17–1.23 | 122 | 4.2 | 1.11 | 1.10–1.13 |
| **Smoker Status** | | | | | | | | | | | | |
| Never | 1,176 | 31.7 | ***1.00*** | — | 447 | 5.9 | ***1.00*** | — | 111 | 3.0 | ***1.00*** | — |
| Former | 2,281 | 55.1 | 1.03 | 1.02–1.04 | 1,025 | 24.8 | 1.28 | 1.26–1.31 | 226 | 5.4 | 1.35 | 1.32–1.37 |
| Current | 575 | 39.5 | 1.12 | 1.10–1.13 | 241 | 16.2 | 1.35 | 1.32–1.37 | 61 | 4.5 | 1.38 | 1.35–1.41 |
| **Physical activity** | | | | | | | | | | | | |
| Active | 557 | 45.0 | ***1.00*** | — | 202 | 15.5 | ***1.00*** | — | 32 | 2.0 | ***1.00*** | — |
| Moderate | 794 | 49.5 | 1.10 | 1.09–1.12 | 270 | 17.1 | 1.10 | 1.09–1.12 | 60 | 3.6 | 1.80 | 1.78–1.83 |
| Inactive | 2,524 | 54.3 | 1.21 | 1.19–1.23 | 1,141 | 24.5 | 1.58 | 1.55–1.60 | 244 | 4.9 | 2.45 | 2.41–2.48 |

* Controlled for age, sex, and the all variables of family income, immigrant status, ethnicity, and education levels.

### Relationship Between Age and Cardiovascular Comorbidities

Age should impact the cardiovascular comorbidities. In the 65 years and over age group, the cardiac comorbidities were about three- to sevenfold higher as compared to the 12–34 year age group (OR from 3.39–7.86) (Table 4).

### Relationship Between Socioeconomic Status and Cardiovascular Comorbidities

Several socioeconomic indicators were associated with an increased prevalence and risk of comorbidity in a diabetic population. These included having a low income (OR from 1.14–1.85), being nonimmigrants (OR from 1.08–1.35), English origin (OR from 1.01–1.28), and having an education of less than secondary school (OR from 1.23–1.86) (Table 4).

## DISCUSSION

Diabetes mellitus and its comorbidities such as hypertension, heart disease, and stroke share several important characteristics, particularly their association with obesity, physical inactivity, cigarette smoking, and heavy alcohol drinking, and both appear to be modifiable through changing these risk factors[19,20]. Cardiovascular disease is the most important cause of mortality among diabetic patients[21]. Activated immunity and cytokine production lead to insulin resistance and other components

**TABLE 4**
**OR and 95% CI of Awareness of Cardiac Comorbidities Among Diabetics by Socioeconomic Status Among Canadians 12 Years of Age and Over (CCHS, Canada, 2003)***

| | Hypertension | | | | Heart Disease | | | | Stroke | | | |
|---|---|---|---|---|---|---|---|---|---|---|---|---|
| | **n** | **%** | **OR** | **95% CI** | **n** | **%** | **OR** | **95% CI** | **n** | **%** | **OR** | **95% CI** |
| **Family income** | | | | | | | | | | | | |
| High income quartile | 491 | 44.6 | 1.00 | — | 149 | 15.2 | 1.00 | — | 35 | 3.3 | 1.00 | — |
| Upper middle income quartile | 1,069 | 51.5 | 1.15 | 1.13–1.17 | 400 | 19.0 | 1.25 | 1.22–1.27 | 89 | 3.4 | 1.03 | 1.02–1.04 |
| Low middle income quartile | 1,173 | 56.7 | 1.14 | 1.12–1.16 | 544 | 26.7 | 1.75 | 1.72–.78 | 125 | 6.1 | 1.85 | 1.81–1.88 |
| Low income quartile | 746 | 50.9 | 1.27 | 1.25–1.29 | 392 | 28.9 | 1.90 | 1.86–1.95 | 94 | 6.4 | 1.94 | 1.90–1.97 |
| **Immigrant status** | | | | | | | | | | | | |
| Immigrants | 644 | 44.9 | 1.00 | — | 224 | 17.0 | 1.00 | — | 55 | 17.0 | 1.00 | — |
| Nonimmigrants | 3,335 | 49.2 | 1.08 | 1.06–1.10 | 1,465 | 23.2 | 1.35 | 1.32–1.37 | 337 | 23.2 | 1.20 | 1.18–1.22 |
| **Ethnicity** | | | | | | | | | | | | |
| First Nations | 800 | 46.3 | 1.00 | — | 376 | 23.8 | 1.00 | — | 66 | 3.9 | 1.00 | — |
| English origin | 1,058 | 56.7 | 1.22 | 1.20–1.25 | 432 | 24.1 | 1.01 | 1.00–1.02 | 100 | 5.0 | 1.28 | 1.26–1.30 |
| French origin | 920 | 49.2 | 1.06 | 1.04–1.07 | 440 | 25.6 | 1.08 | 1.06–1.10 | 79 | 4.3 | 1.10 | 1.08–1.12 |
| Other origin | 2,344 | 51.5 | 1.11 | 1.10–1.13 | 1,053 | 23.7 | 1.00 | 0.99–1.01 | 213 | 4.4 | 1.13 | 1.11–1.15 |
| **Education Level** | | | | | | | | | | | | |
| Postsecondary | 1,947 | 48.47 | 1.00 | — | 764 | 19.65 | 1.00 | — | 173 | 3.48 | 1.00 | — |
| Secondary | 515 | 50.39 | 1.23 | 1.21–1.26 | 203 | 20.47 | 1.11 | 1.09–1.12 | 56 | 5.62 | 1.60 | 1.58–1.63 |
| Less than secondary | 1,418 | 59.55 | 1.23 | 1.21–1.25 | 680 | 28.57 | 1.55 | 1.52–1.58 | 147 | 6.48 | 1.86 | 1.83–1.89 |

* Controlled for age, sex, and the all variables of risk factors (overweight, heavy alcohol drinking, cigarette smoking, and physical activity).

of the metabolic syndrome, establishing the link between diabetes and arteriosclerosis, which accounts for about 80% of all deaths from diabetes, of which 75% are attributable to cardiovascular disease[22]. The present study examined the prevalence of both shared risk factors for cardiovascular disease and diabetes in a diabetic population, as well as the prevalence and risk of one or more cardiac comorbidities within diabetic patients.

The elevated risk of cardiac comorbidities has been previously reported, but our quantification of risk of these comorbidities using odds ratios is unique among the majority of the literature. It is important to note that while hypertension was the most prevalent comorbidity, risk of heart disease was the most increased as compared to the control group. The prevalence of each comorbidity increased in the diabetic patients as age increased, suggesting that addressing the shared risk factors at a younger age may prevent some of the prevalence in later life.

BMI has been previously associated with both diabetes and cardiovascular disease[23,24,25,26, 27,28]. This study demonstrates a clear link between the development of cardiac comorbidities within a diabetic population when the subjects BMI is greater than 25. This is important in that it provides a quantitative link between diabetes, BMI, and cardiovascular disease, which reaffirms the importance of risk factor modification, particularly BMI, once diabetes has been diagnosed in order to prevent cardiac comorbidities, which often lead to early death.

Within the diabetic population, people who abstain from alcohol drinking and quit cigarette smoking were at a lower risk of diabetic comorbidities, suggesting again the need to modify this behavior after the onset of diabetes to prevent associated morbidity and mortality.

Both cardiovascular disease and diabetes have been associated with low socioeconomic status (SES)[29,30,31,32]. The current study shows that people with diabetes, who are of low SES, are more likely to develop a diabetic comorbidity as compared to more affluent people with diabetes. The reasons for this reduced risk in affluent populations are unclear and could range from better health care, access to information about risk behavior modification, or other unknown factors.

The current study adds to the literature that links cardiac diseases and diabetes. As well, it demonstrates, quantitatively, the increase in risk that people with diabetes have in developing cardiovascular disease as compared to a normal population. The increase in risk of diabetic comorbidities as a result of shared risk factors suggests that an intervention targeted at those risk factors within a diabetic population may be vital to reduce the high cost of mortality resulting from diabetic complications. As well, it has recently been postulated that diabetes has a multiplicative interaction with traditional cerebrovascular and cardiovascular disease risk factors, which result in an increase in overall vascular risk when additional risk factors for cardiovascular disease are present in the diabetic patient[33].

This study has several limitations including an issue with self-report. Therefore, there may be misreporting in both the case and control population.

### CONCLUSIONS

The adjusted prevalence of diabetes and its comorbid hypertension, heart disease, and stroke in the CCHS-2003 data was significantly higher among those with diabetes compared to those without. Other factors such as age, gender, BMI, lifestyle, family incomes, health service utilization, and socioeconomic status also affected the strength of association between diabetes and resulting comorbidities and complications.

### BIOSKETCHES

**Frank Mo, PhD, MD**, Centre for Chronic Disease Prevention and Control, Population and Public Health Branch, Health Canada. E-mail: Frank_Mo@hc-sc.gc.ca

**Lisa M. Pogany**, **MSc**, Centre for Chronic Disease Prevention and Control, Population and Public Health Branch, Health Canada.

**Felix C.K. Li, MB BS, FRCPC**, Centre for Chronic Disease Prevention and Control, Population and Public Health Branch, Health Canada.

**Howard Morrison**, **MSc, PhD**, Centre for Chronic Disease Prevention and Control, Population and Public Health Branch, Health Canada.
